# An investigation on the drag reduction performance of bioinspired pipeline surfaces with transverse microgrooves

**DOI:** 10.3762/bjnano.11.3

**Published:** 2020-01-03

**Authors:** Weili Liu, Hongjian Ni, Peng Wang, Yi Zhou

**Affiliations:** 1 School of Petroleum Engineering, China University of Petroleum (East China), Qingdao 266580, China; 2 Key Laboratory of Unconventional Oil & Gas Development (China University of Petroleum (East China)), Ministry of Education, Qingdao 266580, China; 3 Department of Chemical and Petroleum Engineering, University of Calgary, Calgary T2N1N4, Canada

**Keywords:** bionic pipeline, drag reduction, drag reduction mechanism, fluid transport, transverse micro-grooves

## Abstract

A novel surface morphology for pipelines using transverse microgrooves was proposed in order to reduce the pressure loss of fluid transport. Numerical simulation and experimental research efforts were undertaken to evaluate the drag reduction performance of these bionic pipelines. It was found that the vortex ‘cushioning’ and ‘driving’ effects produced by the vortexes in the microgrooves were the main reason for obtaining a drag reduction effect. The shear stress of the microgrooved surface was reduced significantly owing to the decline of the velocity gradient. Altogether, bionic pipelines achieved drag reduction effects both in a pipeline and in a concentric annulus flow model. The primary and secondary order of effect on the drag reduction and optimal microgroove geometric parameters were obtained by an orthogonal analysis method. The comparative experiments were conducted in a water tunnel, and a maximum drag reduction rate of 3.21% could be achieved. The numerical simulation and experimental results were cross-checked and found to be consistent with each other, allowing to verify that the utilization of bionic theory to reduce the pressure loss of fluid transport is feasible. These results can provide theoretical guidance to save energy in pipeline transportations.

## Introduction

With the gradual emergence of an energy crisis [1], it is extremely urgent to improve energy efficiency. In this context, drag reduction is important for vehicle and fluid transportation to increase the cruising speed and to decrease the consumption of energy of these processes. In pipeline transportation, the transport drag originates from skin friction drag, which is the main reason affecting the transport efficiency of long-distance pipelines [2–3]. In drilling engineering, the high pressure loss often encountered is mainly caused by a skin friction drag of the circulating drilling fluid, which severely hinders the exploration of oil and gas resources in deep wells [4–6]; therefore, it is necessary to put additional effort into reducing the skin friction drag.

Conventional hydraulic drag reduction methods include the development of high-performance polymer additives to reduce fluid viscosity [7–9], the injection of gas to modify the turbulent boundary characteristics [10], and the fabrication of superhydrophobic surfaces to reduce the adhesion [11]. However, the application of polymer additives is not economic and the antidrag performance of additives is also instable under some complex conditions. Besides, these active antidrag methods require extra energy or may complicate the devices, which limits their application in engineering.

Pressure loss mainly derives from the shear stress of a fluid flowing across the surface of a pipeline. The wall shear stress is expressed as

[1]
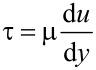


where τ is the shear stress (Pa), μ is the dynamic viscosity of the fluid (Pa∙s); and d*u*/d*y* is the velocity gradient (1/s).

According to Equation 1, changing the turbulent boundary layer state in the vicinity of the wall for a decreased velocity gradient is an essential and appropriate measure to reduce the drag [12]. It is well known that organisms found in nature provide substantial inspiration for solving engineering problems. Bionic research has found that some natural organisms form a specific surface structure with antidrag [13], antiwear [14–15], and hydrophobic [16–17] performance through evolution over billions of years in order to adapt to their environment. Besides, the bionic theories have been applied to many engineering fields, such as microstructured external [18] and internal flow tunnels [19], bionic pump cylinder liners [20], and bionic bearing sliders [21].

**Figure 1:**
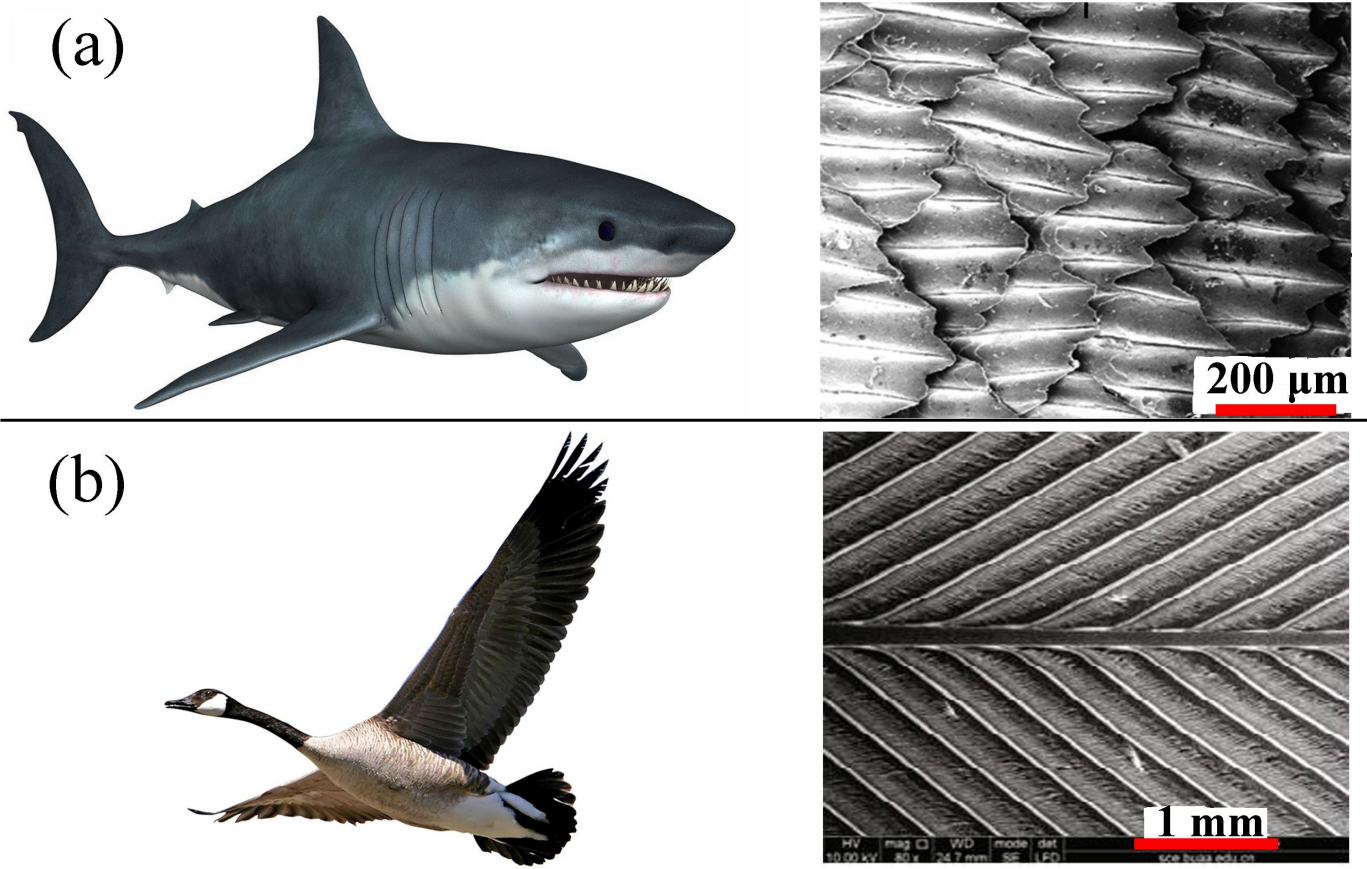
SEM photos of (a) shark skin and (b) bird feather.

Figure 1 shows the surface morphology of shark skin and bird feathers. In Figure 1a, it is evident that the surface of shark skin is rough and covered with microgrooves. Sharks are known to be one of the fastest fish in the ocean. The phenomenon of nonsmooth surfaces with low drag has attracted the attention of researchers. Inspired by shark skin, a method of applying a grooved structure to reduce the drag has been proposed [22]. As can be seen in Figure 1b, bird feathers are also covered with grooves. It has been confirmed that the microgrooves are the crucial factor for the low drag and high speed of shark and bird locomotion [13,22]. Thus, the triangular grooves seen in the nonsmooth structure of animal surfaces have become an effective means to reduce the viscous drag associated with fluid flow. Given the urgent demand to decrease pressure loss in fluid transport and the practicability of bionic theory, the grooved surfaces of shark skin and bird feathers can be imitated and then applied to pipeline surfaces to reduce the viscous drag [23–24]. This provides a novel method to save energy in pipeline transportation.

In the last decades, utilization of bionic microstructures to reduce the drag of turbulent flow has become a research hotspot. This technique allows to save energy in engineering applications [25–26], such as in fuel pipelines, aircrafts, and vehicles. Furthermore, as a passive and portable drag reduction technique, it requires no extra energy. According to the configuration direction of its bionic microstructure, it can be divided into streamwise grooves and transverse grooves. With the development of numerical simulations and experimental techniques, the influence of microstructures on turbulent flow characteristics can be investigated accurately. Its drag reduction mechanism is owed to the two aspects of reducing viscous drag and controlling boundary layer separation [27–28]. By imitating the microgrooves of sharkskin, the drag reduction performance of streamwise grooves has been validated by many researchers, both in external flat and internal rectangular duct turbulent flow systems [18–19,22,29–32], with drag reduction rates (DRR) up to 10%.

Comparatively, research on the drag reduction of transverse microgrooves started late, and their drag reduction performance is controversial. Walsh [33] measured the drag of convex transverse grooves in an external flat flow, and a slightly increased drag rate was found. Tokunaga [34] investigated a turbulent channel flow with one concave transverse groove by the large eddy simulation (LES) method, and the results showed that the turbulence was weakened, and substantial drag reduction was found at the Reynolds number 8000. Dou and co-workers [35] mimicked fish scales to fabricate a bionic surface through coating technology and obtained a remarkable drag reduction performance in a water tunnel experiment. Feng and co-workers [13] mimicked bird feathers to fabricate a bionic surface with transverse grooves through hot-rolling technology and obtained significant drag reduction efficiency in a wind tunnel experiment. Mariotti and co-workers [36] assessed the drag reduction performance of boat-tailed axisymmetric bodies with transverse grooves, with the consequence of significant DRRs owing to the delay of boundary layer separation.

According to the above analysis, most of the previous studies focused on external flow with bionic microstructures. However, there are few studies on the internal flow of pipelines with transverse microgrooves [35]. Therefore, it is necessary to evaluate the drag reduction performance of transverse microgrooves with respect to internal pipe flow, which can provide theoretical guidance for energy saving aspects in fluid pipeline transportation.

In this study, the microgrooved structures of shark skin and bird feathers were simplified to ideal triangular grooves and applied to a pipeline surface. Then, the drag reduction performance of the bionic pipeline with respect to pipe flow and the concentric annulus flow was investigated. Firstly, the computational fluid dynamic (CFD) method was used to analyze the hydraulic characteristics of the pipeline’s inner and outer surface with transverse microgrooves. Besides, the influence of the microgrooves’ geometric parameters on the turbulent flow field and an optimal microgroove structure were obtained. Then, comparative experiments between microgrooved and smooth surfaces were conducted in a water tunnel annular flow to evaluate the drag reduction performance of transverse microgrooves. Finally, the drag reduction mechanism of transverse microgrooves was revealed systematically.

## Methods

### CFD simulation method and validation

#### Computational models

Pressure loss mainly derives from fluid turbulent flow in the pipeline and the annulus between coaxial pipelines, so the inner and outer wall of pipelines with transverse microgrooves need to be modeled. In order to reduce computational workload, two-dimensional axisymmetric models were used to simulate the pipe flow and the concentric annulus flow.

**Figure 2:**
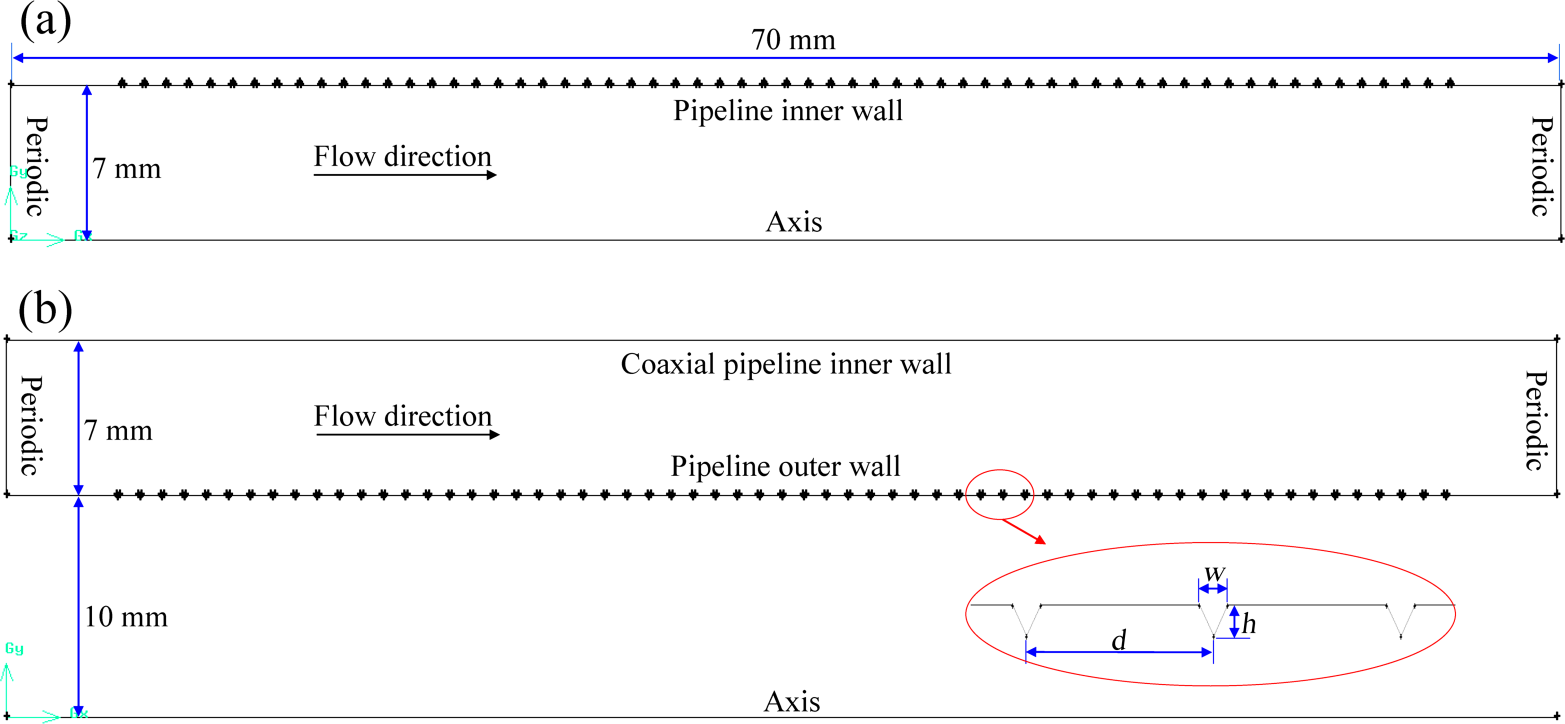
Computational models. a) Pipe flow model with transverse microgrooves. b) Concentric annulus flow model with transverse microgrooves.

The circulating flow of drilling fluid in drilling engineering is a typical annulus flow. Meanwhile, reducing the circulating pressure consumption of drilling fluid is of great significance to improve the efficiency of oil and gas exploration. Therefore, the typical ratio of the outer diameter of the drill string to the inner diameter of the borehole was selected to determine the dimensions of the annular pipe. The annulus’ inner and outer diameter were set to 20 and 34 mm, respectively. The hydraulic diameter of the pipe and annulus were set to *D* = 14 mm, and the flow direction length of the model to *L* = 5 × *D* = 70 mm, according to the suggestion by Eggles [37]. The computational models of pipe flow and annulus flow formed by two coaxial pipelines are shown in Figure 2, transverse microgrooves with triangular sections evenly distributed on the surface of the pipeline. The model of a smooth wall with the same diameter and length was also established for comparison of its flow drag with the bionic pipeline under the same condition.

#### Governing equations

A commercial CFD tool, ANSYS Fluent-18.0, was used to calculate the turbulent flow in the pipe and the concentric annulus. Near-wall turbulence characteristics of a drag-reducing polymer fluid flow in the concentric annulus was successfully simulated with the shear stress transfer (SST) κ–ω model [38]. The SST κ–ω model was developed by Menter to render it independent from the κ–ε model in a wide range of fields. Besides, the SST model combined the advantages of the κ–ε model (near-wall) and κ–ω model (away from the wall), which reduced the influence of wall grid density on computational accuracy. This is why the SST κ–ω model has been widely used to solve the turbulent flow field. Considering the computational accuracy and cost, the Reynolds-averaged Navier–Stokes (RANS) numerical simulation method with the SST turbulence model was adopted to analyze the hydraulic characteristics of the near-wall turbulent flow. The governing equations were as follows:

Continuity equation:

[2]
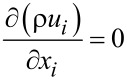


Momentum equation:

[3]
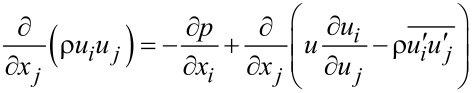


κ equation:

[4]
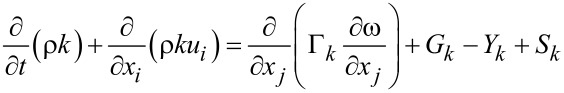


ω equation:

[5]
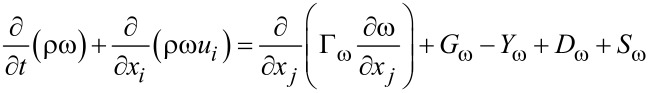


where *i*, *j* were the coordinate direction and direction of the velocity components, *i* = 1,2,3, *j* = 1,2,3; *u_i_*, *u_j_* were the speed of different coordinate directions (m/s); ρ was the density of fluid (kg/m^3^); 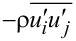 was the Reynolds stress; and *G_k_* was the turbulent kinetic energy produced by the velocity gradient. Further, *G_w_* was generated by the ω equation; Γ*_k_*, Γ_ω_ were the effective diffusion terms of *k* and ω; *Y_k_*, *Y*_ω_ were the turbulences generated by diffusion; *D*_ω_ was the orthogonal divergence term; and *S_k_*, *S*_ω_ were user-defined terms.

#### Boundary conditions and other parameters settings

In order to compare the hydraulic characteristics of a grooved and a smooth pipeline and to exclude the effects of other factors on the simulation results, the calculations used the same parameter settings, and the specific conditions were set as follows: (1) The periodic boundary condition was adopted along the flow direction to guarantee the turbulent flow was fully developed. The no-slip boundary condition was adopted for the wall (as shown in Figure 2). The desired velocity was achieved by adopting a steady mass flow boundary condition. (2) Incompressible water was used as continuous phase medium. The density was 998.2 kg/m^3^ and the dynamic viscosity was 0.001003 Pa∙s. (3) The pressure-velocity coupling scheme was SMIPLEC. The momentum equation was discretized by the second-order upwind scheme to ensure accuracy and stability.

#### Independence validation of grid density

**Figure 3:**
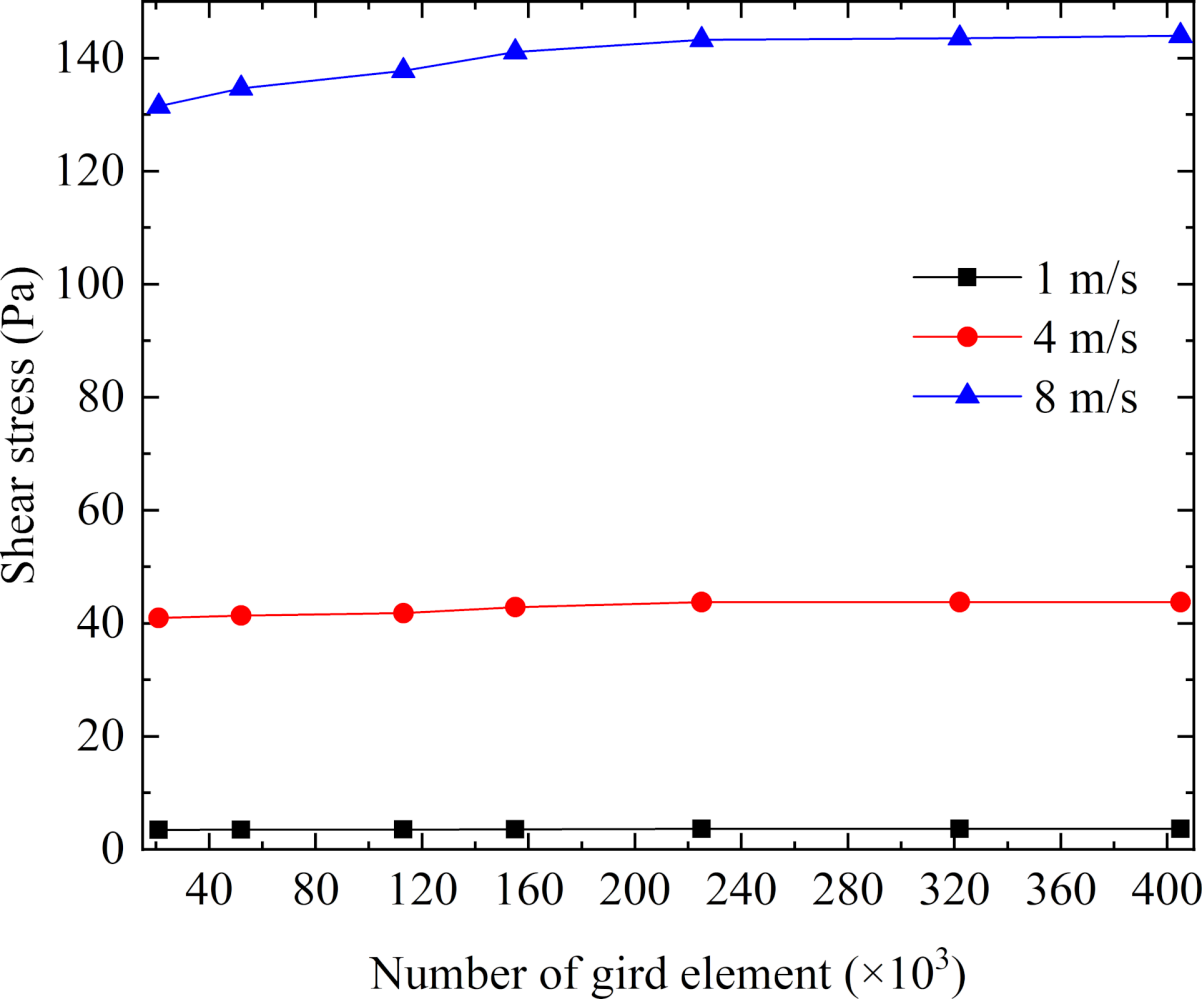
Influence of grid density on the viscous drag of the pipe flow.

The finite volume method was used to discretize the computational domain to unstructured triangular grids. The grids of the grooved wall were refined, and the grids became gradually coarse by a size function as they were further away from the wall. Grid density would affect the computational accuracy, so the influence of the grid density on the shear stress by using a smooth pipeline was analyzed. As can be seen in Figure 3, when the number of grid elements exceeded 320000, the shear stress tended to be stable at different velocities. Therefore, the number of grid elements should not be less than 320000 in the numerical simulation.

**Figure 4:**
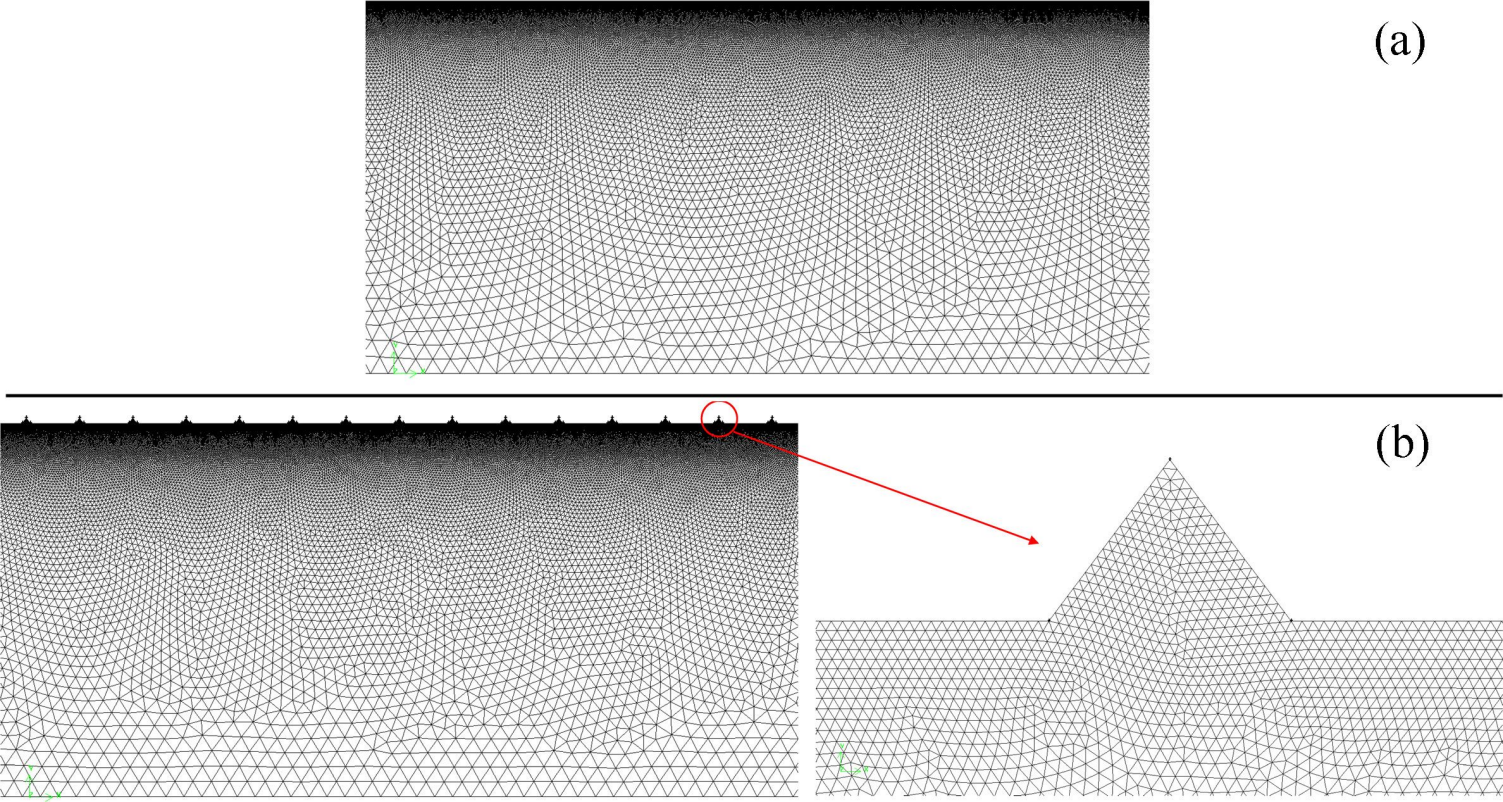
Computational grids of the a) smooth pipeline and b) microgrooved pipeline.

The smooth and bionic models were meshed with the same meshing strategy to guarantee that the computational results were independent from the grid density. The computational grids are depicted in Figure 4.

#### Validation of CFD results

Before analyzing the turbulent flow, it was essential to validate the accuracy of the numerical simulation methods.

**(1) Validation of the pipe flow:** The simulated viscous coefficient of the smooth pipeline was compared to the results when using the empirical formula. The relevant formulas were expressed as follows [39]:

Reynolds number:

[6]
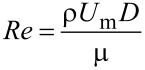


The empirical formulas for the viscous coefficient of a smooth pipeline were as follows:

Braustian formula:

[7]
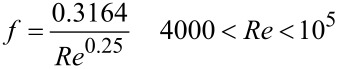


Nikolaze formula:

[8]
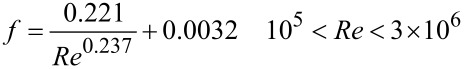


Numerical simulation for the viscous coefficient of a smooth pipeline:

[9]
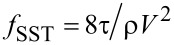


where *U*_m_ was the bulk velocity (m/s); *D* was the hydraulic diameter (m); and τ was the shear stress (Pa), which was calculated by a numerical simulation. As can be seen from the comparison data in Table 1, the maximum relative error was within the allowed range. Therefore, the present numerical simulation method was reliable.

**Table 1:** Comparison of empirical and simulated viscous coefficients of the pipe flow.

*U*_m_ (m/s)	*Re*	*f*	*f*_SST_	relative error (%)

1	13933	0.02912	0.02941	−1.01
5	69665	0.01948	0.02011	−3.18
10	139300	0.01654	0.01699	−2.72
20	278660	0.01452	0.01436	1.11

**Figure 5:**
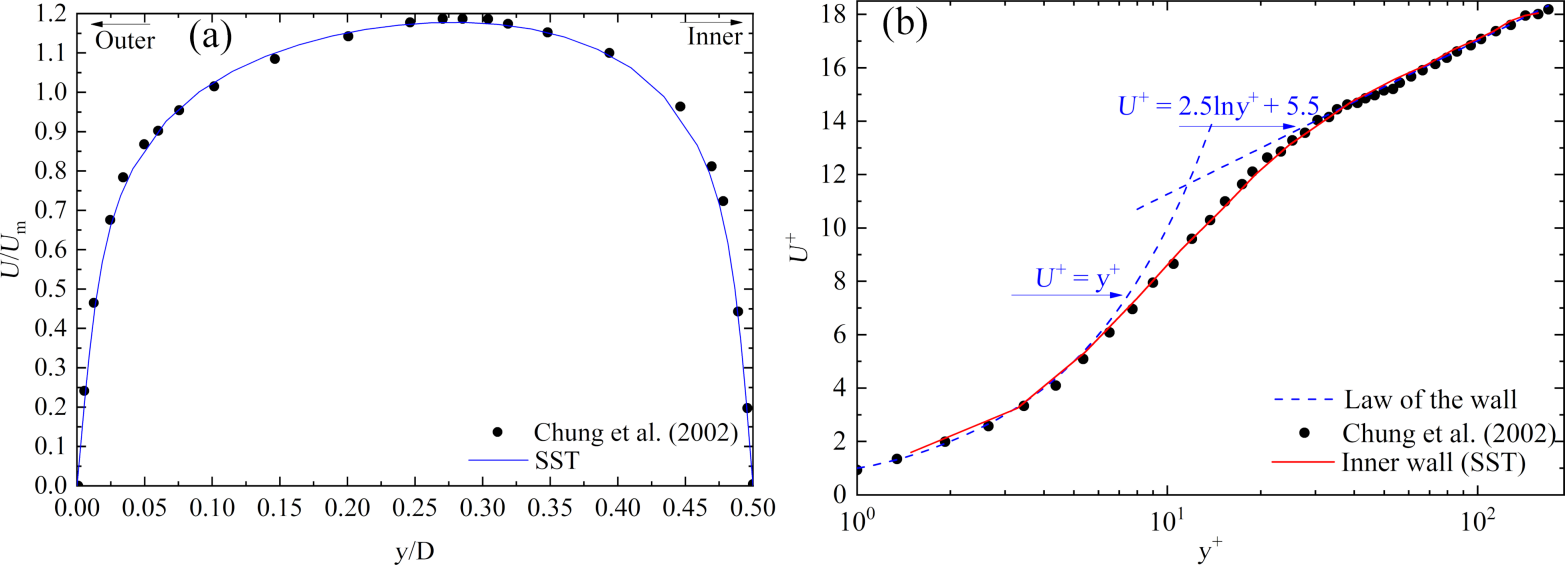
Comparison of SST results with DNS results. a) Mean velocity distribution of the annulus flow. b) Velocity distribution for the law of wall.

**(2) Validation of the annulus flow:** Next, the annulus flow with identical boundary conditions as used by Chung and co-workers [40] was calculated by the present numerical simulation method. The velocity distributions of the annulus flow were compared to the direct numerical simulation (DNS) results, as shown in Figure 5. The agreement with DNS was satisfactory so that the accuracy of the numerical simulation method could be validated again.

Dimensionless parameters:

[10]
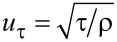


[11]
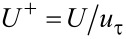


[12]
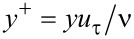


where *u*_τ_ was the friction velocity (m/s); *U* was the instantaneous velocity (m/s); *y* was the normal distance from the wall (m); and *ν* was the kinematic viscosity (m^2^/s).

#### Drag reduction evaluation method

Under the same simulation conditions, the average drag of the smooth and the bionic pipeline were compared to obtain the DRR of transverse microgrooves, which was defined as follows:

[13]
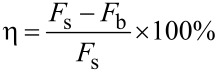


where η was the DRR (%); *F*_s_ and *F*_b_ were the average drag of the smooth and the bionic surface (N). *F*_b_ included the viscous and pressure drag. A positive DRR value indicated that the transverse microgrooves had a drag reduction effect.

### Experimental method

#### Experimental setup and procedure

**Figure 6:**
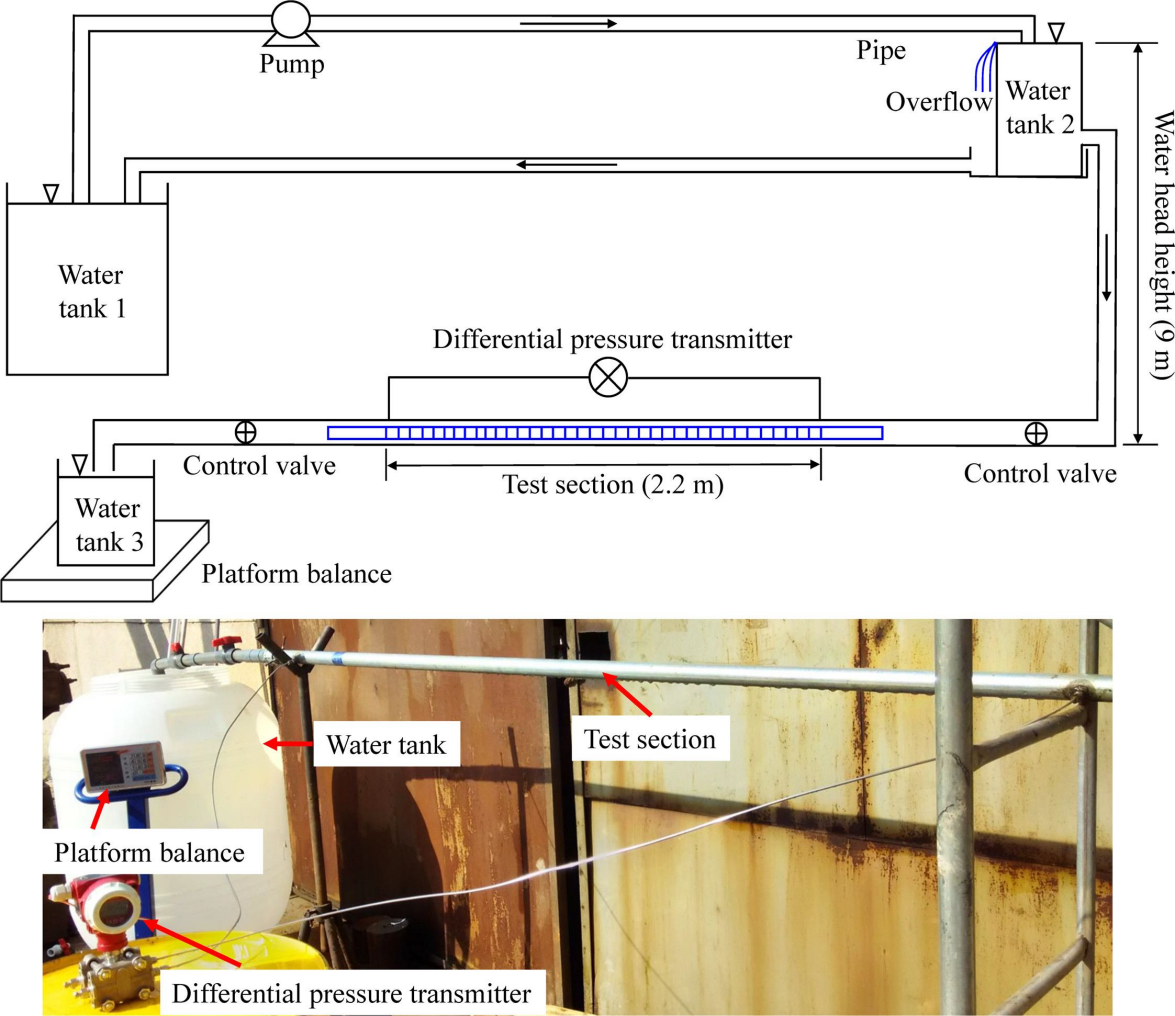
Schematic diagram of the experimental setup.

The accuracy and reliability of the numerical simulation results needed experimental verification, so comparative experiments of bionic and smooth pipelines were carried out in a water tunnel. Figure 6 shows the pressure loss testing setup for a concentric annulus flow. The length of the test section was 2.2 m, the circulation medium was tap water, and the flow rate could be changed by a control valve. During the entire experimental procedure, the liquid level of water tank 2 remained at overflow to guarantee a consistent water head height for the contrast experiment. In order to ensure that the test section was in a fully developed turbulent flow, the length of the annulus entrance section and export section were reserved with 0.5 m.

Accurate measurement of pressure loss and flow rate in the test section were the key to evaluate the hydraulic characteristics of the pipeline surface. The pressure loss of the annulus test section was measured by a differential pressure transmitter (DMP305X-DST) based on monocrystalline silicon technology. The monosilicon pressure transmitter was a high-performance pressure sensor. Its measuring range was 0–20000 Pa, and its precision was ±0.05%. Considering that the electromagnetic flow meter had a large measuring error at high flow rate conditions, the flow rate was measured by the weighing method. The measuring range and precision of the platform balance were 0–500 kg and ±0.01%.

The experimental procedures were as follows: Firstly, the scaled pipeline was installed and positioned in the center of the annulus. Secondly 30 groups of pressure drop values in intervals of 5 s were gathered to obtain the average value after the flow was stable. Thirdly, the water of water tank 3 collected for 120 s was weighed to calculate the flow rate. The average values of pressure loss and flow rate were measured in triplicate experiments under the same conditions to decrease the measuring error.

#### Specimen preparation

**Figure 7:**
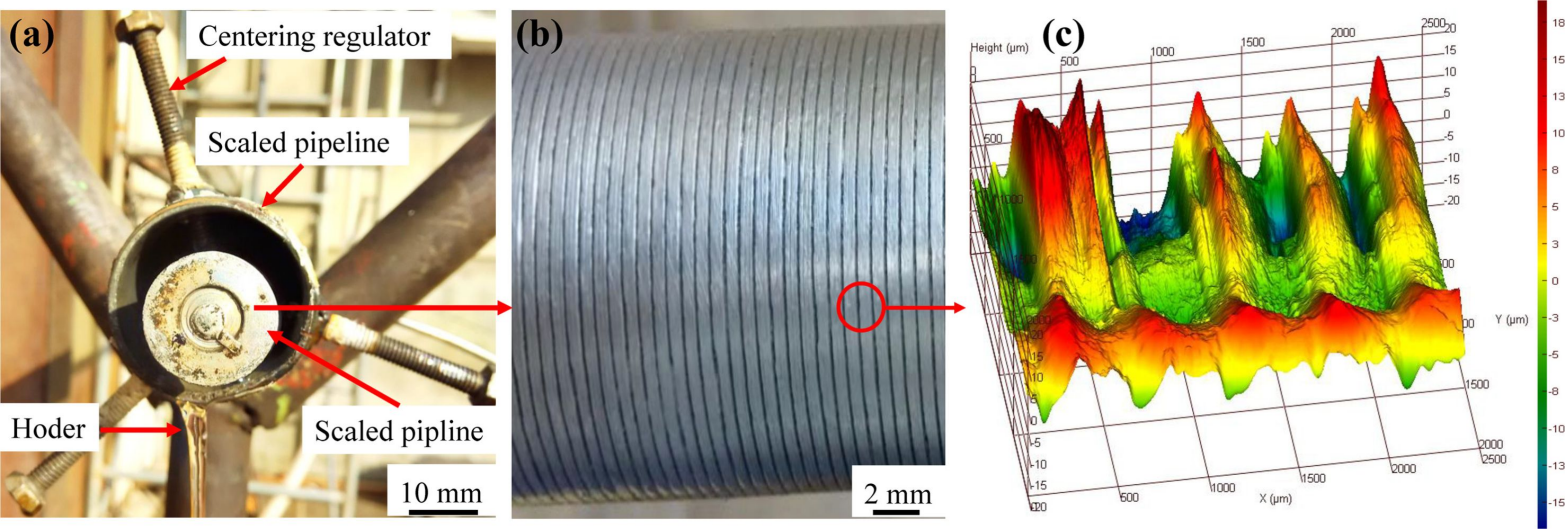
Specimen. a) Scaled and b) grooved pipeline in a concentric annulus flow. c) Micromorphology of the microgrooves.

It is difficult to machine microgrooves on the inner surface of a pipeline due to restrictions by processing conditions, so the hydraulic characteristics of the pipeline’s outer surface in a concentric annulus flow were tested exclusively. As can be seen from Figure 7a, the annulus channel was composed of aluminum pipelines with different diameters. The outer diameter of the inner scaled pipeline was 19.5 mm, the inner diameter of the outer scaled pipeline was 35 mm, and the inner pipeline was fixed by the centering regulators. Based on the microgrooves’ geometric parameters obtained by the numerical simulation, microgrooves were manufactured on the outer surface of the aluminum pipeline by lathe machining, as shown in Figure 7b. The height and width of the microgrooves could be controlled by the shape of the lathe tool, and the distance between the grooves could be controlled by a screw pitch. The micromorphology of the microgrooves is illustrated in Figure 7c.

#### Validation of experimental reproducibility and accuracy

**Figure 8:**
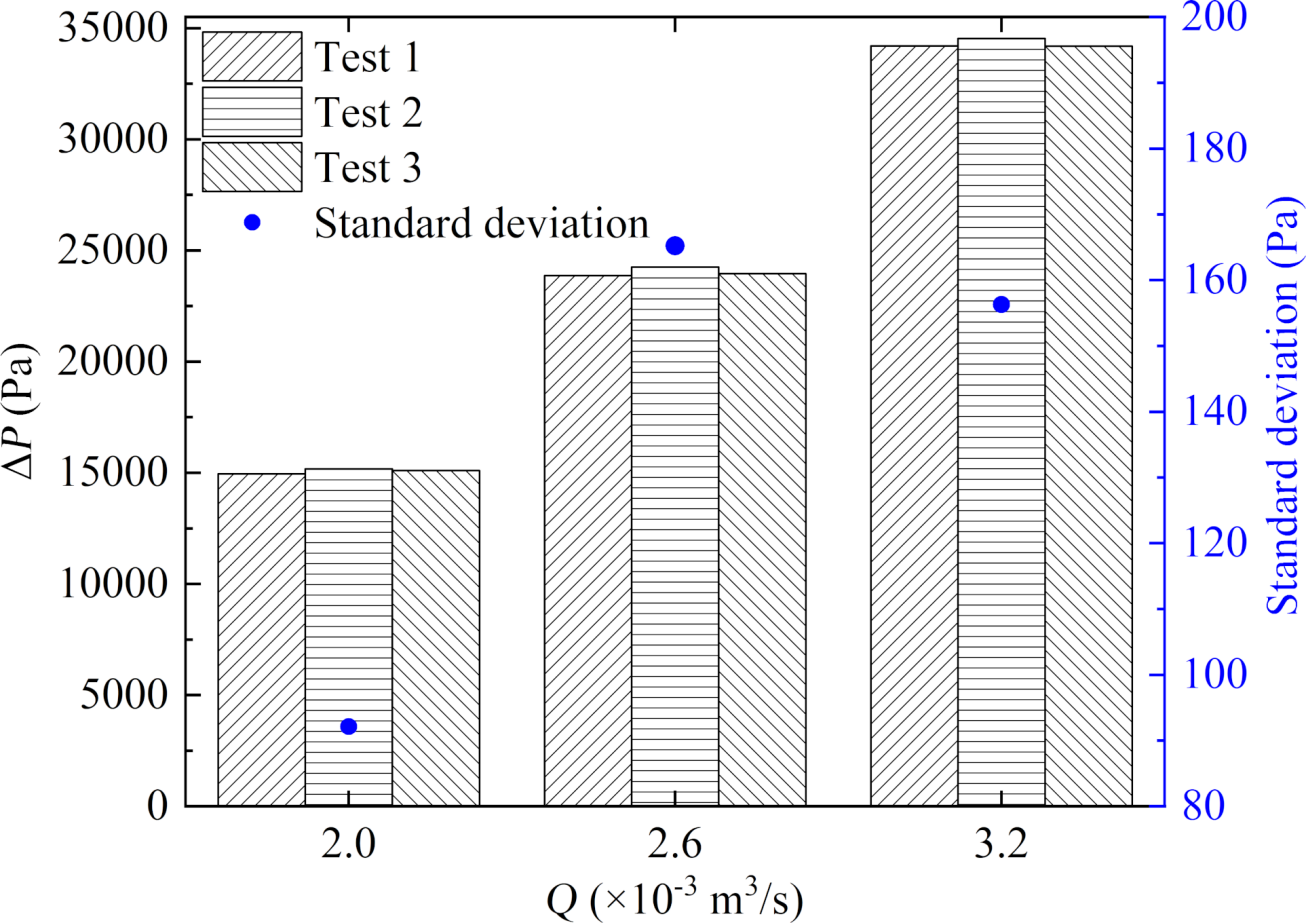
Comparison of triplicate experimental results in an annulus flow.

Firstly, triplicate experiments were carried out under the same conditions to verify the reproducibility of the experimental method. As can be seen in Figure 8, the results of the three experiments were approximately consistent, and the standard deviation was acceptable compared to the magnitude of the pressure difference.

**Figure 9:**
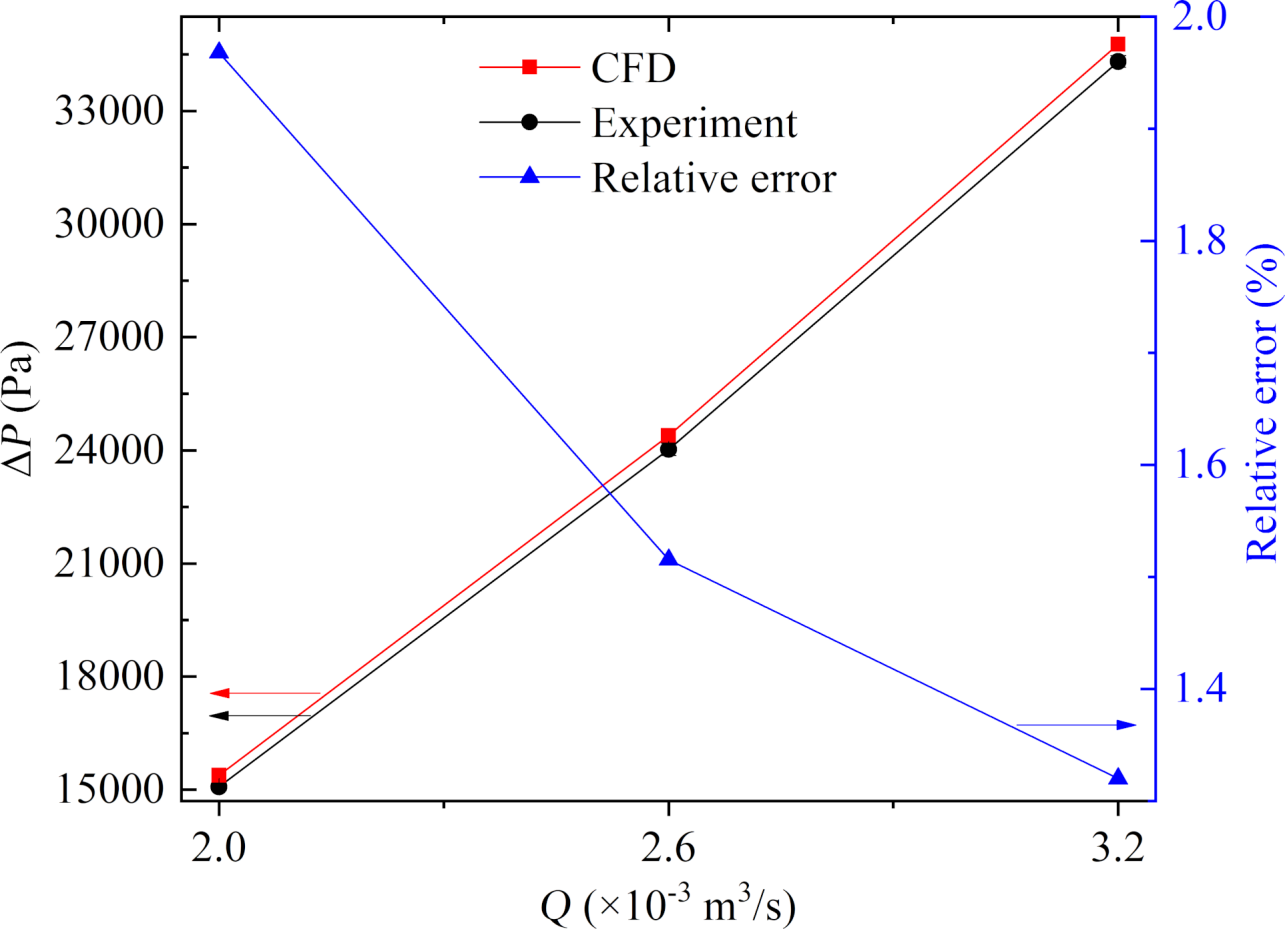
Comparison of average pressure differences obtained from numerical simulations and experiments.

In addition, the measured average pressure difference was compared to the result of numerical simulations at the same flow rate. Figure 9 shows the comparison of the simulated and experimental results, and the maximum relative error was less than 2.0%, which was deemed acceptable. Therefore, it could be concluded from the above analysis that the experimental method was accurate, with good reproducibility at different flow rates.

#### Drag reduction evaluation method

Under the same experimental conditions, the average frictional head loss of the smooth pipeline and the bionic pipeline were compared to obtain the DRR of the pipeline with transverse microgrooves, which could be defined as follows:

[14]
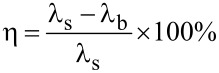


where λ_s_ and λ_b_ were the friction loss factors of the smooth and the bionic pipeline.

The frictional head loss of the smooth and the bionic pipeline could be calculated by the following equations:

Smooth pipeline:

[15]
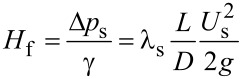


Bionic pipeline:

[16]
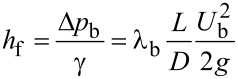


Flow rate:

[17]
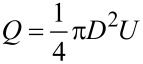


Substituting Equations 15–17 by Equation 14, the DRR of the bionic pipeline was given by:

[18]
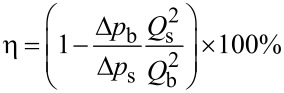


where *Q* was the flow rate (m^3^/s); Δ*p*_s_ and Δ*p*_b_ were the pressure difference of the smooth pipeline and the bionic pipeline (Pa); *L* was the length of the test section (m); and γ was the unit weight of water (N/m^3^). A positive DRR indicated that the transverse microgrooves had a drag reduction effect in the concentric annulus flow setup.

## Results and Discussion

### Single-factor sensitivity analysis of groove geometric parameters

As shown in Figure 2, the groove height *h*; the groove width *w*, and the distance between the grooves *d* were three significant geometric parameters of the microgrooves, which determined the drag reduction effect of the transverse microgrooves. The influence of the microgrooves’ geometric parameters on the drag reduction performance was analyzed by the single factor analysis method.

#### Microgroove height

**Figure 10:**
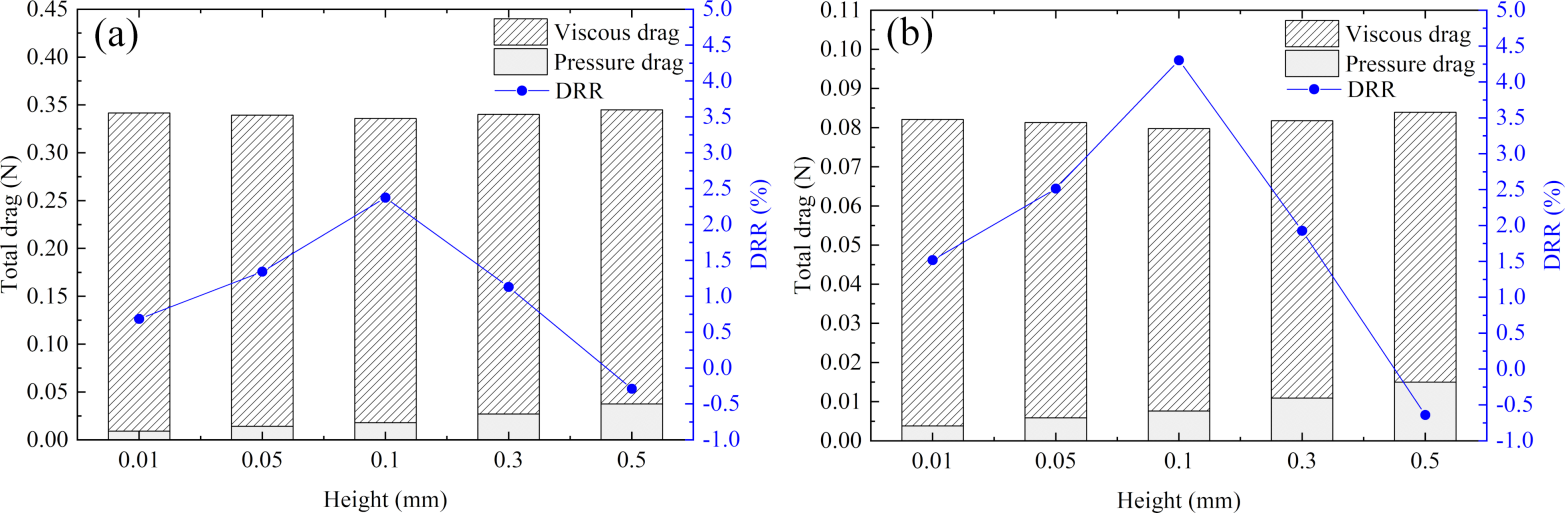
Effect of microgroove height on the drags in a) pipe flow and b) an annulus flow systems.

Firstly, the values *w* = 0.2 mm, *d* = 1 mm, and *Re* = 50000 were kept constant. The flow drag and DRR of grooves with different heights in the pipe and in an annulus flow are shown in Figure 10a and Figure 10b, respectively. From Figure 10a and Figure 10b, the transverse microgrooves had both advantages and disadvantages with respect to the flow drag. The decreasing viscous drag was beneficial to achieve an effective DRR, while extra pressure drag was detrimental to the drag reduction performance. In case the reduction in viscous drag was greater than the increase in pressure drag, the microgrooves will had a drag reduction effect. The groove height had the same effect both in the pipe and in an annulus flow: the pressure drag increased with the increase in height, and the DRR first increased and then decreased. At the same time, the DRR of the annulus flow was lower than that of the pipe flow because the microgrooves could only affect the inner wall of the annulus.

#### Microgroove width

**Figure 11:**
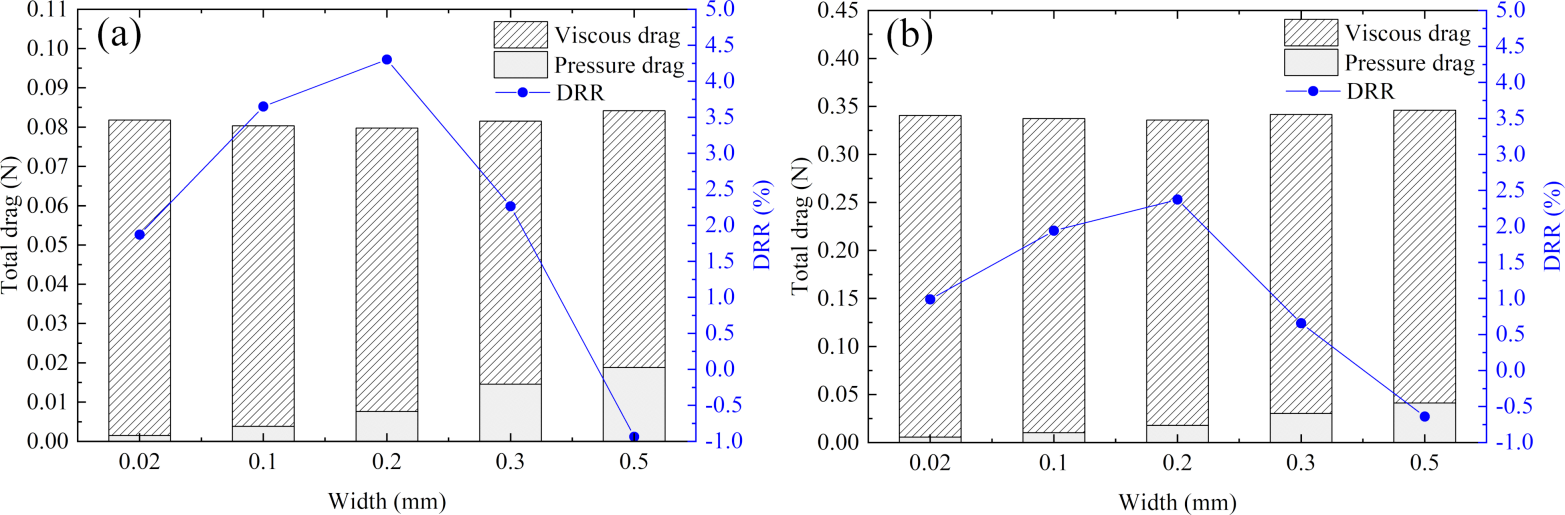
Effects of microgroove width on the drags in a) pipe flow and b) annulus flow systems.

Secondly, the values *h* = 0.1 mm, *d* = 1 mm, and *Re* = 50000 were kept constant. The flow drag and DRR of grooves with different widths in the pipe and in an annulus flow are shown in Figure 11a and Figure 11b, respectively. From Figure 11a and Figure 11b, it can be seen that the grooves’ width had the same influence on the drag in the pipe and under annulus flow conditions: the pressure drag increased with increasing width, and the DRR first increased and then decreased.

#### Distance between microgrooves

**Figure 12:**
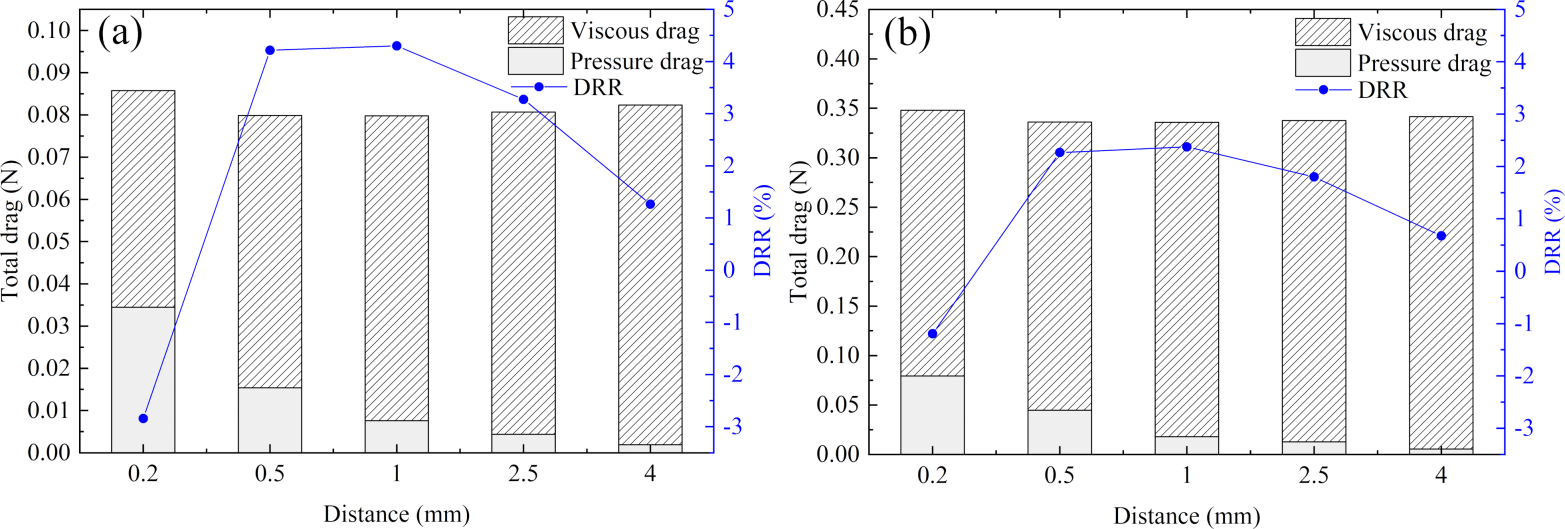
Effect of microgroove distance on drags in a) pipe flow and b) annulus flow systems.

Finally, the values *h* = 0.1 mm, *w* = 0.2 mm, and *Re* = 50000 were keep constant. The flow drag and DRR of grooves with different distances in the pipe and in an annulus flow are shown in Figure 12a and Figure 12b, respectively. From Figure 12a and Figure 12b, it can be seen that the grooves’ width had the same influence on the drag in the pipe and under annulus flow conditions: the pressure drag decreased with increasing distance. Meanwhile, the viscous drag increased as the distance increased, which led to a DRR that first increased and then decreased.

#### Optimization of the microgrooves’ geometric parameters

The geometric parameters of grooves had a significant effect on the drag reduction performance, based on the analysis of single factors. It was necessary to optimize the parameters to further improve the DRR of grooves. In order to investigate the interaction between height, width, and distance of grooves on the drag reduction effect, and to select the optimal parameters, an orthogonal table of L_9_(3^4^) was adopted for the simulation of parameter design (as shown in Table 2). The simulation results were processed by the intuitive analysis method using the DRR as evaluation index.

**Table 2:** Grooves’ parameters optimization by orthogonal analysis in a pipe flow model.

test number		factor A	factor B	factor C	evaluation index
	
		*h* (mm)	*w* (mm)	*d* (mm)	DRR (%)

1		0.05	0.1	0.5	4.03
2		0.05	0.15	1	5.78
3		0.05	0.2	2	4.55
4		0.1	0.1	1	4.84
5		0.1	0.15	2	5.62
6		0.1	0.2	0.5	4.40
7		0.15	0.1	2	2.69
8		0.15	0.15	0.5	3.89
9		0.15	0.2	1	3.56
level	k_1_	4.787	3.853	4.107	
	k_2_	4.953	5.097	4.727	
	k_3_	3.380	4.170	4.287	
range		1.573	1.244	0.620	
optimal parameters		0.1	0.15	1	6.26

Table 2 shows the detailed simulation parameters and orthogonal analysis results of the pipe flow system at a flow velocity of 5 m/s. The primary and secondary order of effect on DRR were height, width, and distance, and the optimal microgroove parameters were found to be *h* = 0.1 mm, *w* = 0.15 mm, and *d* = 1 mm. Hence, a maximum DRR of 6.26% could be achieved using the optimal microgroove parameters.

**Figure 13:**
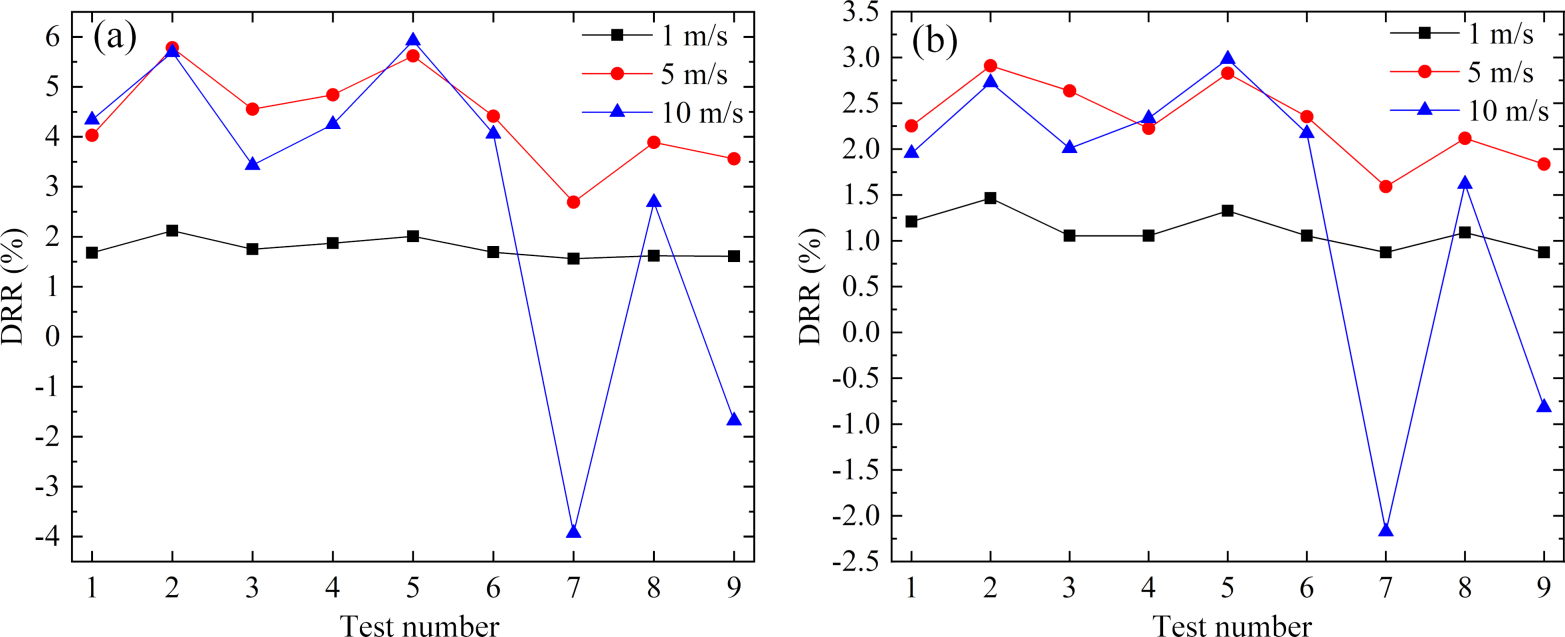
DRR obtained from different numerical tests versus velocity in a) pipe flow and b) annulus flow systems.

By using the same method, orthogonal simulated calculations were carried out under different velocities both in the pipe and in an annulus flow. Figure 13 shows the DRR of different numerical tests at flow velocities of 1 m/s, 5 m/s, and 10 m/s. From Figure 13, it can be seen that the flow velocity had the same influence on the DRR in both the pipe and the annulus flow system. At low velocity, the influence of the microgroove parameters on the DRR was small; with increasing flow velocity, the DRR of each numerical test was significantly different. That is to say, as the Reynolds number increased, the influence of the microgroove parameters on the drag reduction became more sensitive to the effects of drag reduction owing to a decreasing thickness of the near-wall viscous sublayer.

As shown in Table 3, the primary and secondary order of geometric parameters in the pipe and in an annulus flow were the same, also at different flow velocities. The optimal parameters, however, changed slightly at a velocity of 10 m/s. As the velocity increased, the optimal width decreased, which was beneficial for entrapping the vortexes in the microgrooves.

**Table 3:** Orthogonal test range analysis.

velocity (m/s)	primary and secondary order		optimal parameters	

	pipe flow	annulus flow	pipe flow	annulus flow

1	A > B > C	A > B > C	A_2_B_2_C_2_	A_2_B_2_C_2_
5	A > B > C	A > B > C	A_2_B_2_C_2_	A_2_B_2_C_2_
10	A > B > C	A > B > C	A_2_B_1_C_2_	A_2_B_1_C_2_

#### Effect of the Reynolds number on the drag reduction in an annulus flow

From the orthogonal test, it was found that the flow velocity had a significant effect on the drag reduction, and the same influence on the pipe and the annulus flow system. Therefore, the effect of the Reynolds number on the DRR of bionic pipelines with optimal microgroove parameters was only analyzed in an annulus flow using a broader range of Reynolds numbers.

**Figure 14:**
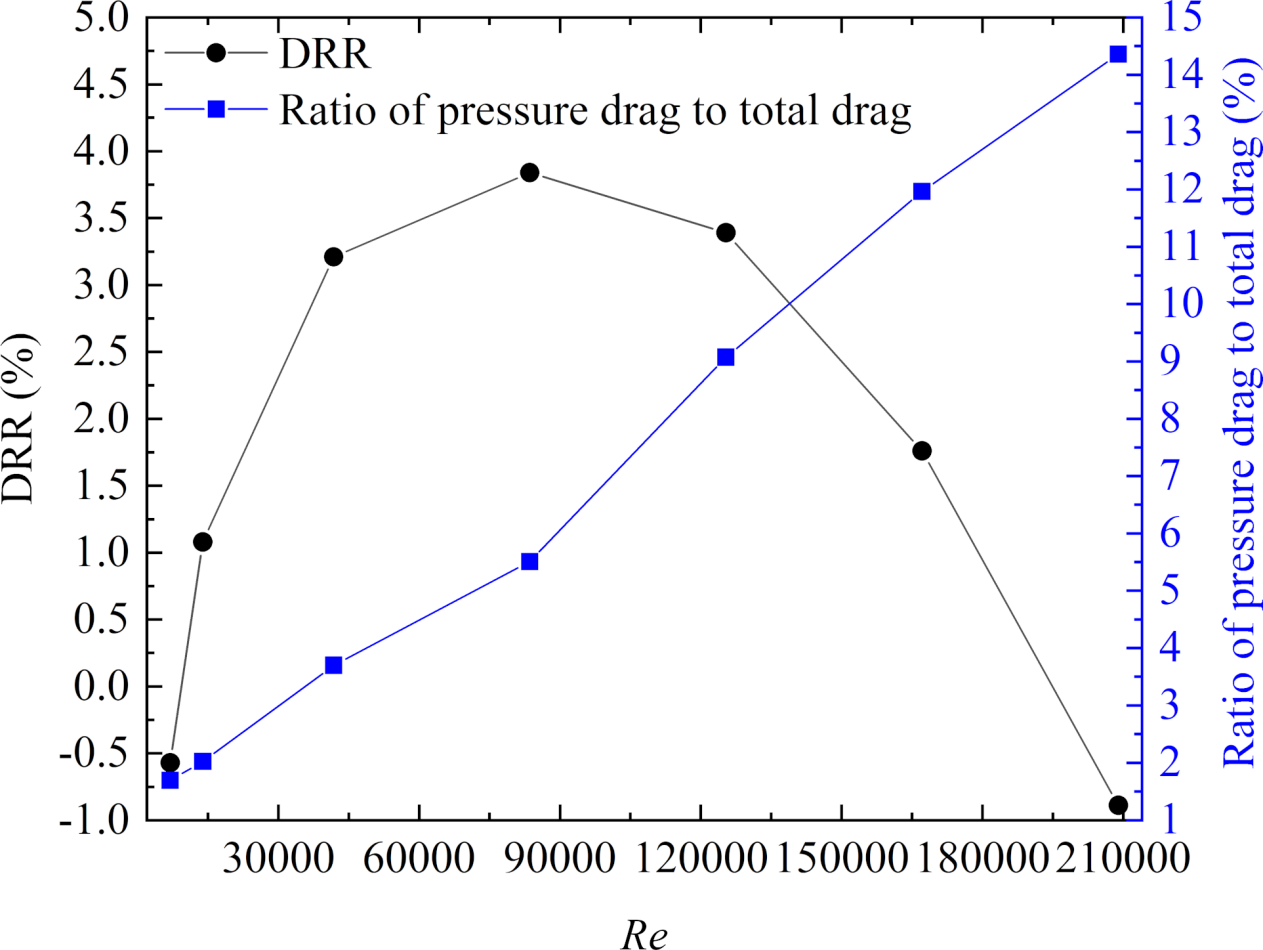
DRR and ratio of pressure drag to total drag versus *Re* in an annulus flow.

Figure 14 shows the influence of the Reynolds number on the DRR and the ratio of pressure drag to total drag. From Figure 14, it can be seen that the DRR first increased and then decreased with an increasing Reynolds number. Besides, the bionic pipeline obtained a maximum DRR of 3.84% when the Reynolds number was 83500. At a low Reynolds number, stable vortexes could not be formed in the microgrooves due to a lack of energy, which was a crucial factor for drag reduction, resulting in the transverse microgrooves increasing the drag slightly. In addition, the ratio of pressure drag to total drag increased rapidly as the Reynolds number increased. Therefore, with the increase of flow velocity, the increase of pressure drag could exceed the reduction in viscous drag, and the microgrooves lost their drag reduction effect eventually.

#### Experimental validation of the microgroove drag reduction performance

**Figure 15:**
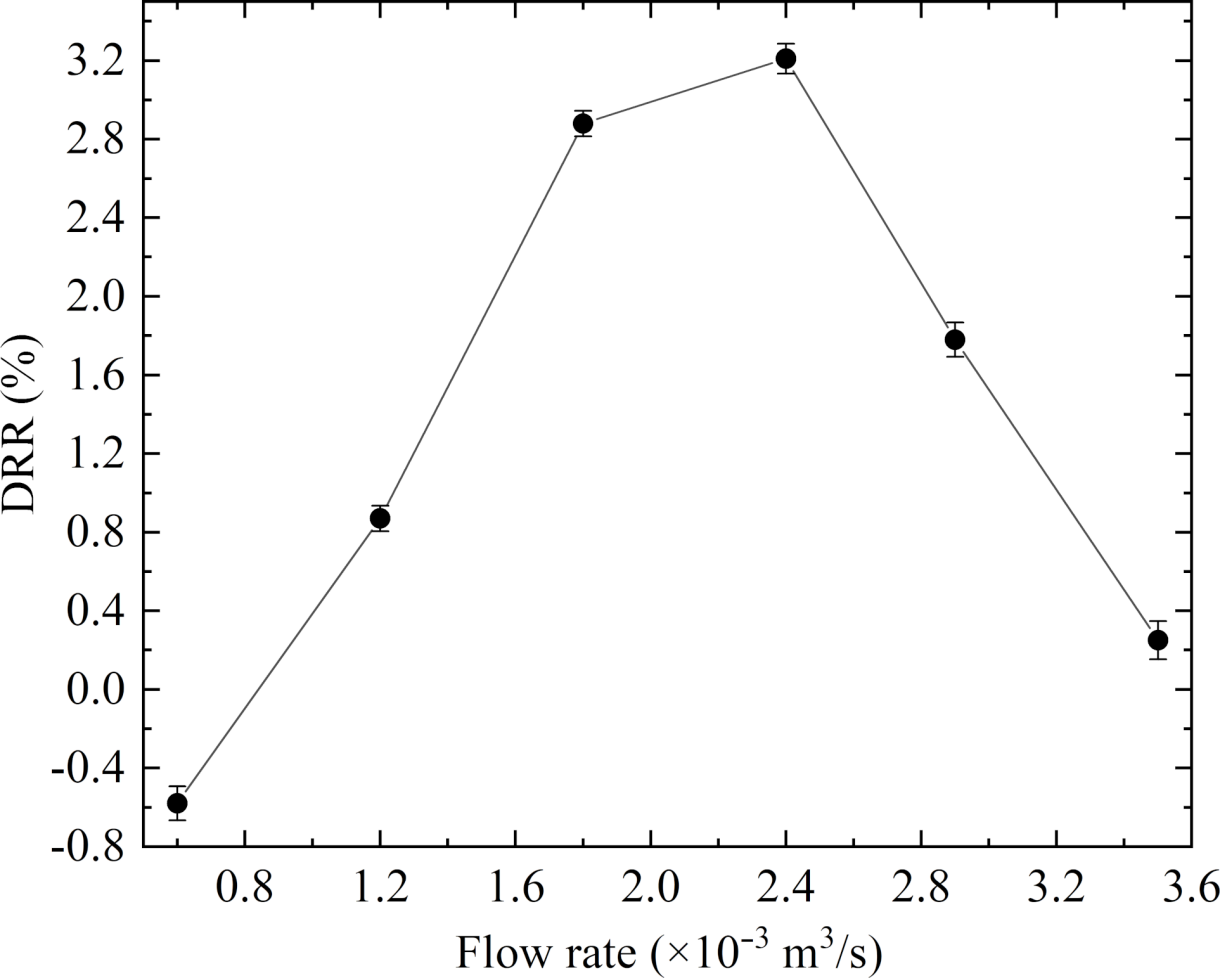
Influence of the flow rate on the DRR in an annulus flow.

In an experimental study, microstructures were machined on the outer surface of the scaled pipeline using the optimal microgroove parameters obtained from the numerical simulation. Then, the frictional drag coefficient of the bionic pipeline was compared to that of the smooth pipeline in the annulus flow at different flow rates. The DRR of the microgrooved pipeline versus the flow rate is plotted in Figure 15. The trend of the curve shows a good agreement with the simulation results. The DRR first increased and then decreased with the increase of flow rate, and the bionic pipeline had a drag reduction effect within a certain flow rate range.

For the annulus flow, the maximum DRR obtained in the experiments was 3.21%, which was lower than the maximum DRR of 3.84% obtained in the numerical simulations. Meanwhile, the experimental Reynolds number range associated with a drag reduction effect was smaller than that obtained in the numerical simulation (Table 4). The different results may have been caused by specimen machining errors. As illustrated in Figure 7c, it was inevitable to produce small burrs around the grooves in the machining process, which was limited by processing accuracy and processing conditions. In the numerical simulation, the computations used ideal microgrooves with an absolutely smooth surface. As can be seen in Figure 7c, the effect of the burrs was to increase the surface roughness of the pipeline. Besides, the greater the surface roughness was, the higher the pressure loss of fluid flow was. Therefore, in the experiment, the burrs on the pipeline surface caused pressure loss, which resulted in the maximum DRR of the experiment being slightly lower than the simulated one.

The circulating flow of drilling fluid in drilling engineering is a typical example of pipeline fluid transport. Meanwhile, reducing the circulating pressure consumption of drilling fluid is of great significance to improve the efficiency of oil and gas exploration. Therefore, the feasibility of microgrooves for drag reduction in drilling fluid circulation is discussed hereafter.

According to the numerical and experimental results, the Reynolds number was a crucial factor affecting the drag reduction of a microgrooved surface. As can be seen in Table 4, the approximate Reynolds number range of commonly used drilling fluid in drilling sites [6] was included in the Reynolds number range associated with a drag reduction effect. Therefore, it should theoretically be feasible to reduce the circulating pressure loss in drilling engineering by using microgrooved drill pipes. However, the feasibility will need to be verified by experiments in further studies.

**Table 4:** Approximate Reynolds number range of commonly used drilling fluid in drilling sites.

*Re* range with positive DRR		*Re* range of drilling fluid

simulation	experiment	ρ ≈ 1200–1800 kg/m^3^; µ ≈ 0.02–0.04 Pa∙s; *Q* ≈ 0.03–0.06 m^3^/s; *D* ≈ 0.1 m
*Re* ≈ 8000–160000	*Re* ≈ 15000–90000	*Re* ≈ 11000–75000

#### Flow field characteristics and drag reduction mechanism

Taking pipe flow as an example, the drag reduction mechanism was discussed by comparing the flow field characteristics of a smooth and a bionic pipeline with optimal microgroove parameters at a flow velocity of 8 m/s.

#### Flow field characteristics analysis

**Figure 16:**
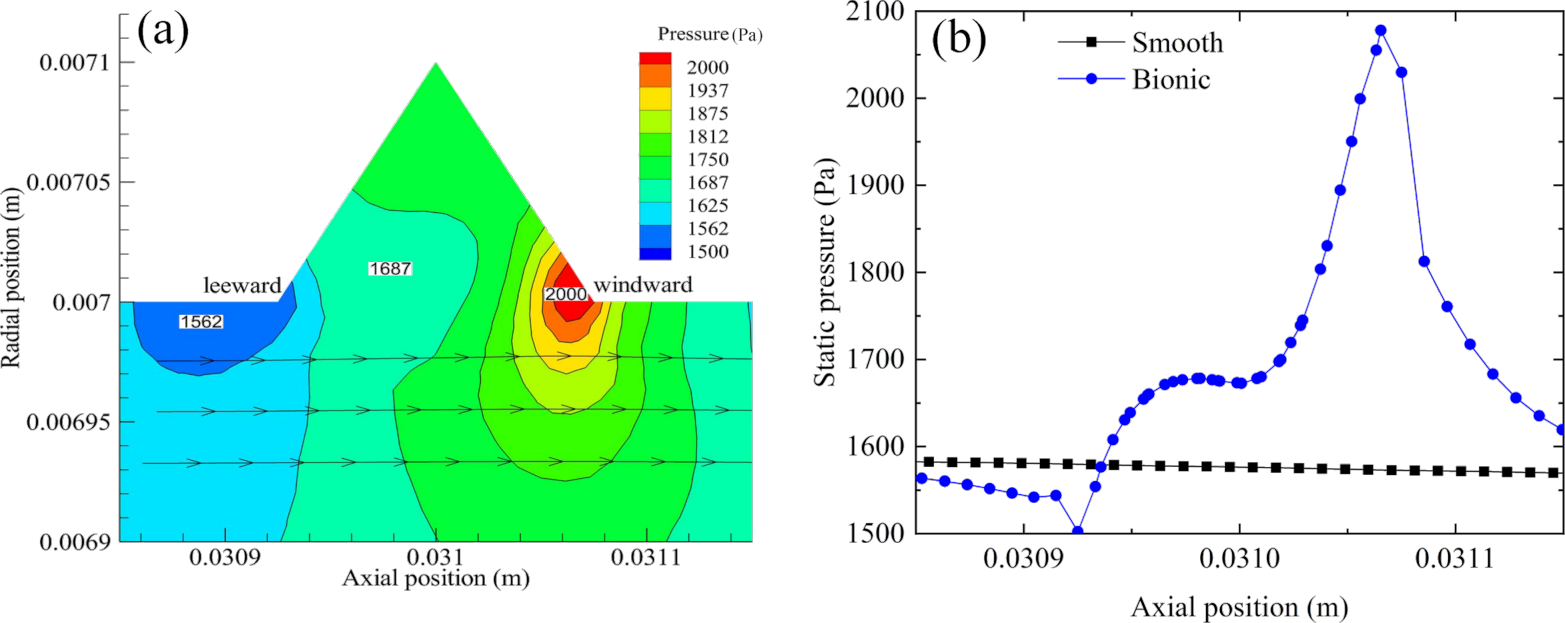
Pressure distribution. a) Pressure contour near the groove. b) Pressure curve at the wall.

**(1) Pressure drag:** The pressure distribution around the near-wall is illustrated in Figure 16, revealing the reason for the extra pressure drag induced by the microgroove. As shown in Figure 16, a local high-pressure zone was formed on the windward of the microgroove, and a local low-pressure zone was formed on the leeward of the microgroove. It was the adverse pressure gradient that caused the pressure drag, which was not conducive to drag reduction.

**Figure 17:**
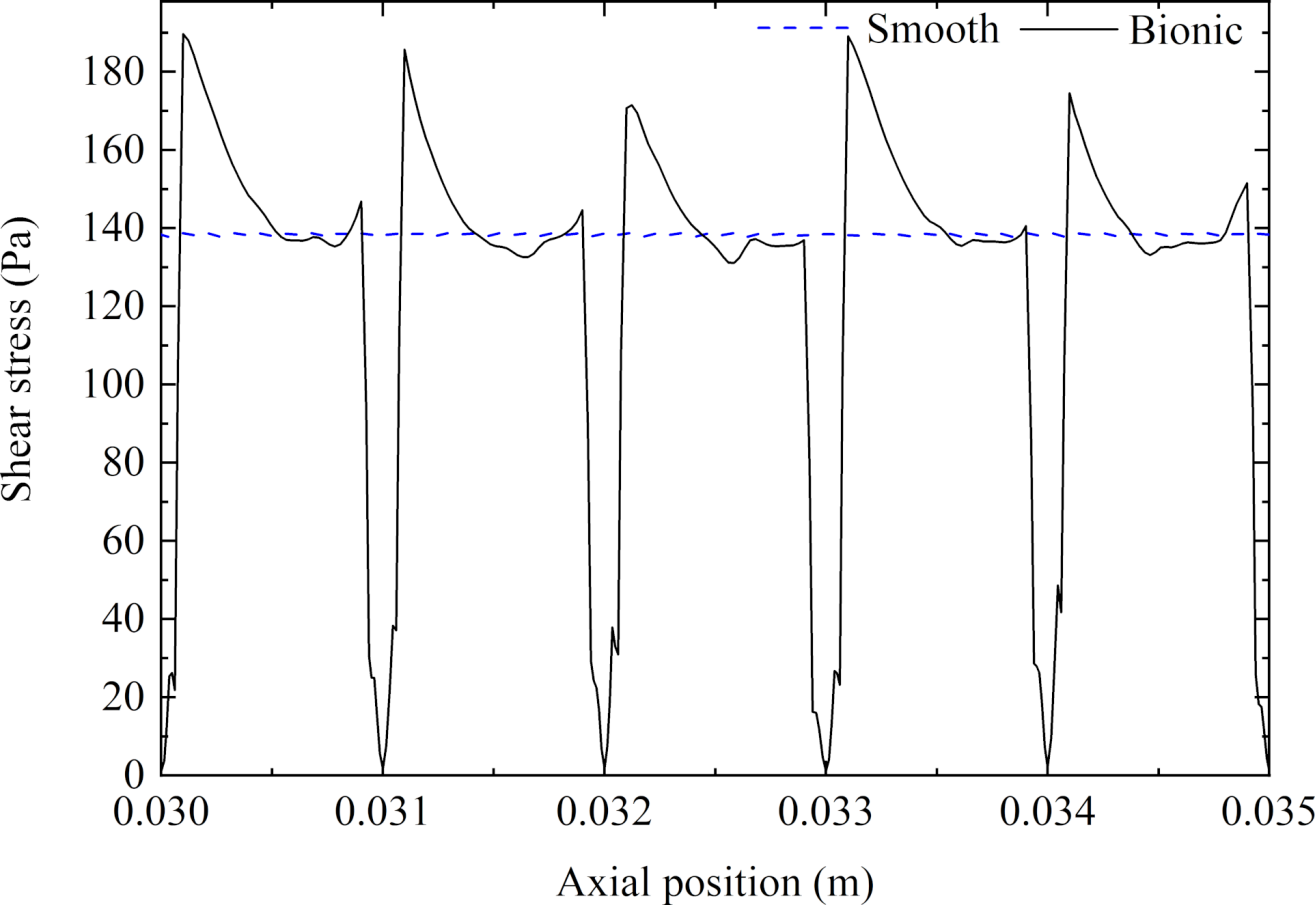
Wall shear stress of a smooth and a bionic pipeline.

**(2) Shear stress:** Figure 17 depicts the wall shear stress of the smooth and the bionic pipeline at the same axial position. In Figure 17, it can be seen that the shear stress of the bionic pipeline first increased sharply on the windward the of microgroove, then decreased quickly, finally being lower than that of a smooth pipeline. In addition, the shear stress for the microgroove was significantly reduced, forming a ‘low valley’ on the curve. The mean shear stress of the bionic surface was lower than that of the smooth surface, which was the main reason for the reduction in viscous drag. Therefore, when the reduction in viscous drag was greater than the increase in pressure drag, the microgroove exerted a drag reduction effect.

**Figure 18:**
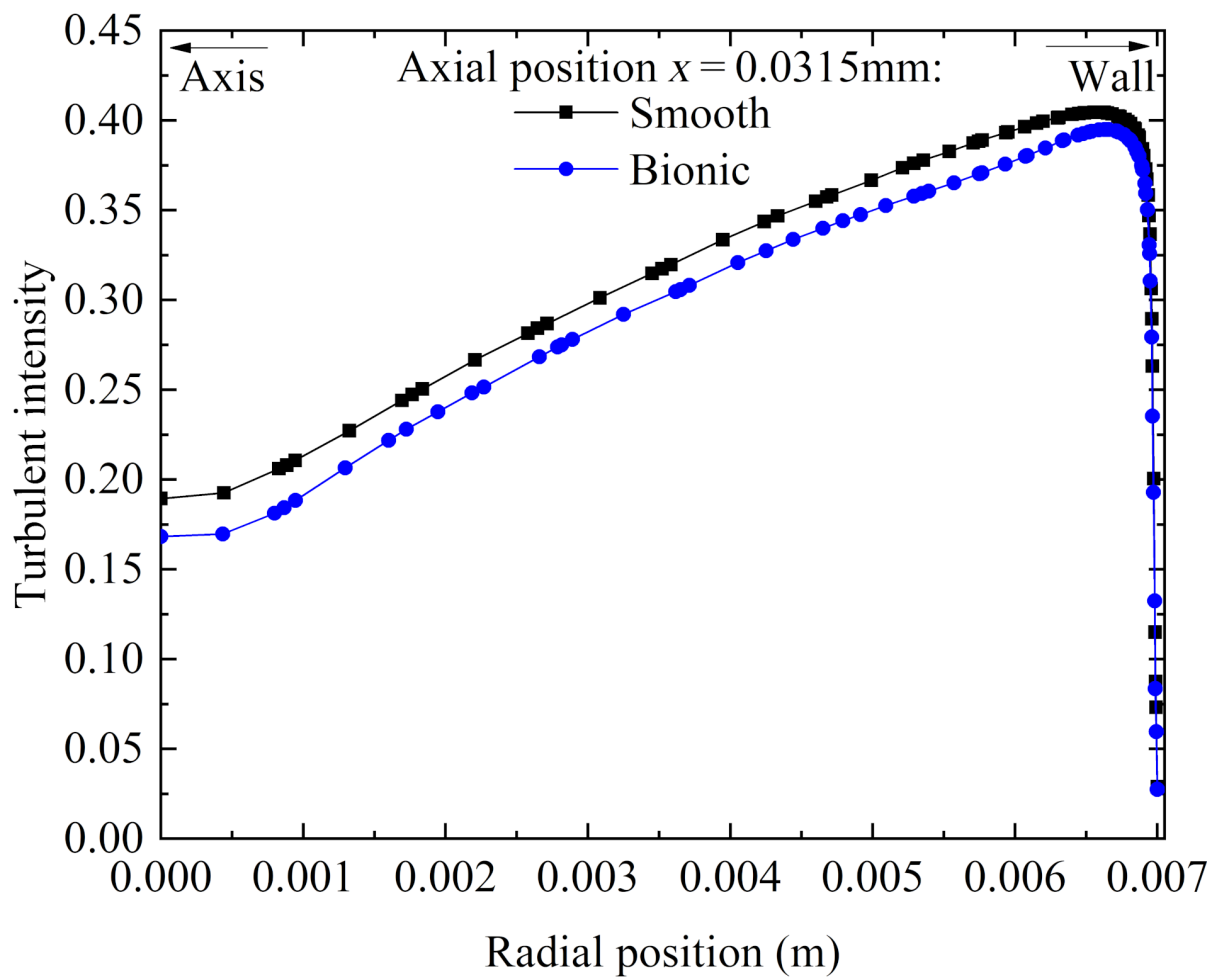
Distribution of turbulent intensity along the radial direction.

**(3) Turbulent intensity:** Turbulent intensity is the ratio of turbulent fluctuating velocity to average velocity, which can intuitively reflect the magnitude of Reynolds stress. Figure 18 shows the distribution of turbulent intensity along the radial direction of the smooth and the bionic pipeline flow field at the same axial position. As can be seen in Figure 18, the turbulent intensity of the bionic pipeline in turbulent transition and core zones was lower than that of the smooth pipeline. Therefore, the transverse microgrooves could reduce the Reynolds stress to achieve a drag reduction effect.

#### Discussion of the drag reduction mechanism

**Figure 19:**
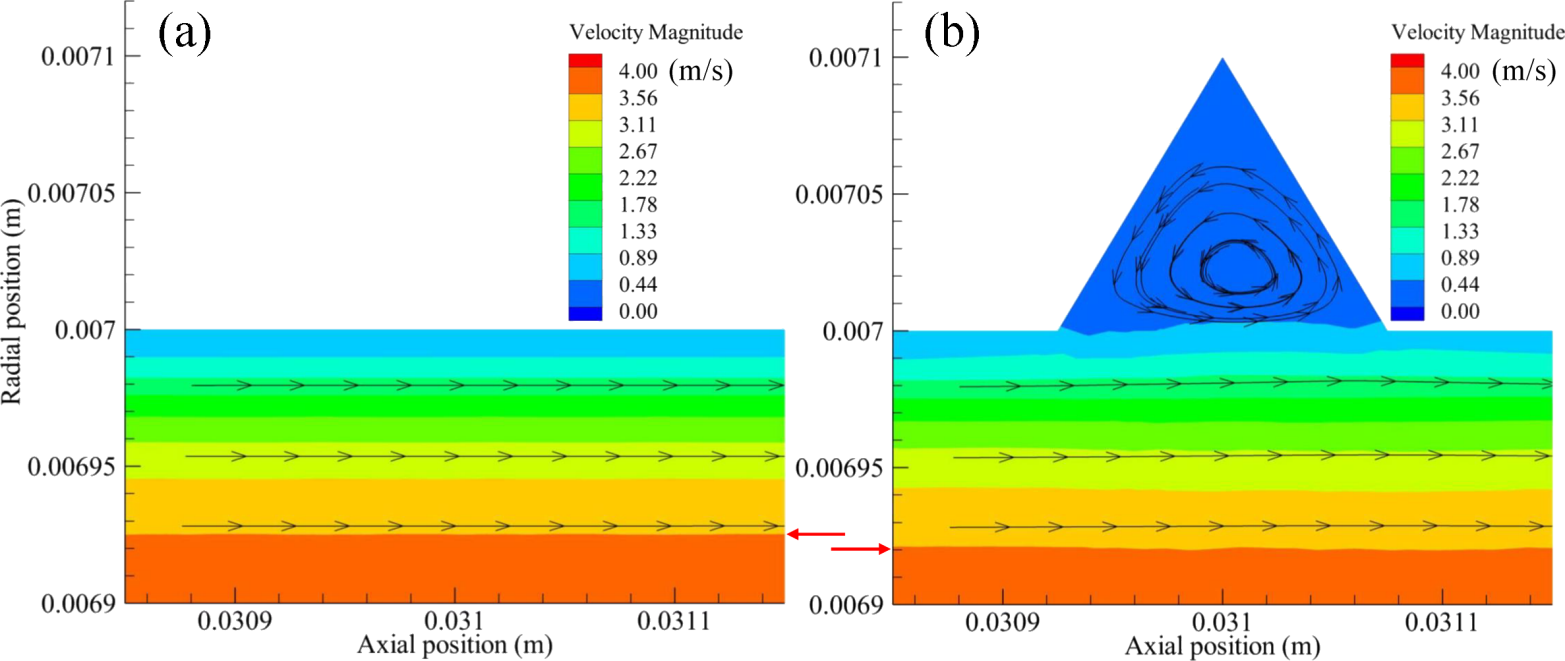
Velocity contours and streamlines near the a) smooth and b) the bionic surface.

**(1) Velocity distribution:** As can be seen from the velocity contours in Figure 19, the boundary layer thickness of the bionic surface was slightly increased compared to the smooth surface. Therefore, the shear stress of the bionic surface was reduced owing to the reduction of the velocity gradient. In Figure 19b, low-speed microvortexes formed in the microgrooves due to the shear action of external fluids. Firstly, the microvortexes gathered in the microgroove to form a vortex ‘cushioning’ effect, like a rolling bearing. Consequently, the external fluid flew through the microgrooves without contacting the solid wall. The sliding friction between the solid and the liquid interface in the smooth pipe flow changed into rolling friction between two liquid interfaces in the bionic pipe flow. In addition, extra frictional drag formed between the vortexes and grooved wall, whose direction was the same as the flow direction. This resulted in the frictional drag having a ‘driving’ effect on the fluid flow. As such, the reduction of viscous drag was mainly caused by the above effects.

**Figure 20:**
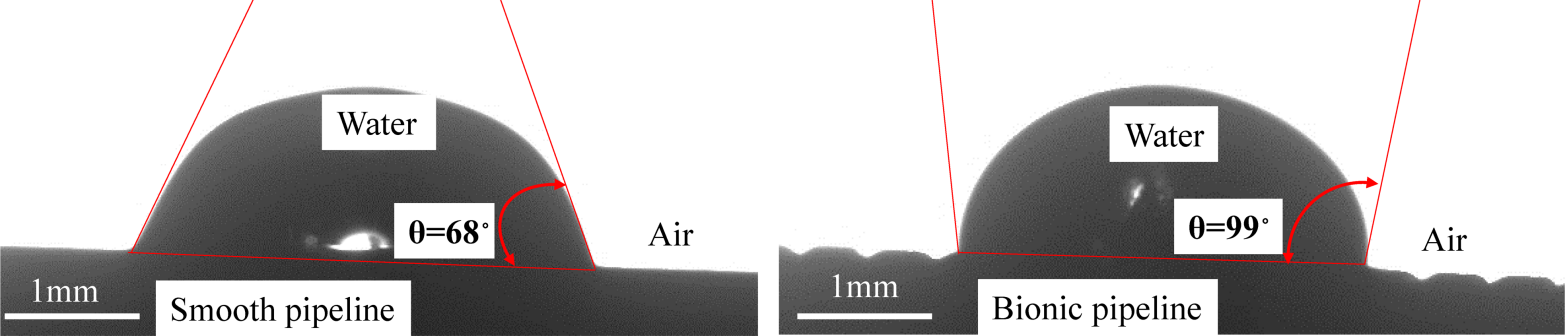
The profile of a droplet on the smooth and the bionic surface.

**(2) Wettability:** Jung and co-workers [41] found through experiments that microstructures with a higher contact angle provided a higher reduction of pressure drop in both laminar and turbulent water flows. Since surface wettability had a significant influence on the turbulent flow drag, the contact angle of the experimental specimen was measured by the sessile drop method to investigate the impact of the microgrooves on the wettability of the pipeline surface. As shown in Figure 20, the contact angles were 68 and 99° on the smooth and the microgrooved surface. In this case, the hydrophobicity of the pipeline surface was improved by the transverse microgrooves. The better the hydrophobicity of the surface was, the lower the adhesion of water was. Therefore, the increased contact angle of the bionic pipeline was beneficial for reducing the drag.

## Conclusion

In this study, the feasibility of applying bionic theory to pipeline surfaces to reduce the drag was investigated by numerical simulations and experimental methods. Besides, the drag reduction mechanism of transverse microgrooves was revealed in terms of flow field characteristics and wettability. The following conclusions can be summarized: (1) The transverse microgrooves had the same influence on the pipe and the concentric annulus turbulent flow – reducing the viscous drag and inducing extra pressure drag. The DRR of the annulus flow was lower than that of the pipe flow under the same conditions. (2) The drag reduction performance was significantly affected by the microgrooves’ geometric parameters. The primary and secondary order of effect on DRR were height, width, and distance, and the optimal parameters were *h* = 0.1 mm, *w* = 0.15 mm, and *d* = 1 mm. This conclusion was the same for the pipe and the annulus flow. (3) The pipeline with appropriate microgrooves had a drag reduction effect at a certain flow rate, and a maximum DRR of 3.21% was achieved in the annulus flow experiment at a flow rate of 2.4 × 10^−3^ m^3^/s. (4) The CFD model predictions were in good agreement with the experimental results in terms of the influence of the Reynolds number on the DRR. The DRR first increased and then decreased with an increasing Reynolds number. The Reynolds number range with drag reduction effect was approximately 15000–90000. Therefore, it should theoretically be feasible to reduce the pressure loss in fluid transport by bionic pipelines. (5) The drag reduction mechanism was mainly attributed to the vortex ‘cushioning’ and ‘driving’ effects, which were produced by low-speed vortexes in the microgrooves. In addition, the hydrophobicity of the pipeline surface was improved by the transverse microgrooves, which could reduce the adhesion of water.

In the present study, the proposition of applying bionic theory to the surface of a pipeline to reduce the drag was verified from a theoretical perspective under ideal conditions. The influence of microgrooves on the strength of the pipeline and the processing of bionic pipelines need to be further studied from the perspective of engineering application.

## Abbreviations

CFD - computational fluid dynamic; RANS - Reynolds-averaged Navier–Stokes; DNS - direct numerical simulation; DRR - drag reduction rate; SIMPLEC - simple-consistent; LES - large eddy simulation; SST - shear stress transfer.

## Nomenclature

τ - shear stress, Pa; *y* - normal distance from wall, m; μ - dynamic viscosity of fluid, Pa·s; *v* - kinematic viscosity, m^2^/s; d*u*/d*y* - velocity gradient, 1/s; η - drag reduction rate, %; 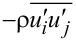 - Reynolds stress; *F*_s_ - average drag of a smooth surface, N; ρ - density of a fluid, kg/m^3^; *F*_b_ - average drag of a bionic surface, N; *L* - length of model, m; λ - friction loss factor; *D* - hydraulic diameter, m; Δ*p* - pressure difference, Pa; *Re* - Reynolds number; *Q* - flow rate, m^3^/s; *U*_m_ - bulk velocity, m/s; γ - unit weight of water, N/m^3^; *f* - viscous coefficient; *h* - height of a microgroove, mm; *u*_τ_ - friction velocity, m/s; *w* - width of a microgroove, mm; *U* - instantaneous velocity, m/s; *d* - distance between microgrooves, mm.
